# Circulating Proprotein Convertase Subtilisin/Kexin type 9 level independently predicts incident cardiovascular events and all-cause mortality in hemodialysis black Africans patients

**DOI:** 10.1186/s12882-022-02748-0

**Published:** 2022-03-30

**Authors:** François-Pantaléon Musungayi Kajingulu, François Bompeka Lepira, Aliocha Natuhoyila Nkodila, Jean-Robert Rissassy Makulo, Vieux Momeme Mokoli, Pepe Mfutu Ekulu, Justine Busanga Bukabau, Yannick Mayamba Nlandu, Augustin Luzayadio Longo, Nazaire Mangani Nseka, Laura Labriola, Ernest Kiswaya Sumaili

**Affiliations:** 1 Department of Internal Medicine, Division of Nephrology, Kinshasa University Hospital, Faculty of Medicine, University of Kinshasa, Kinshasa, Democratic Republic of Congo; 2 Faculty of Family Medicine and Primary care, University of Protestant in Congo, Kinshasa, Democratic Republic of Congo; 3 Department of Pediatrics, Division of Nephrology, Kinshasa University Hospital, Faculty of Medicine, University of Kinshasa, Kinshasa, Democratic Republic of Congo; 4 Université Catholique de Louvain / Cliniques Universitaires Saint Luc de Bruxelles, Brussels, Belgium

**Keywords:** Black Africans, Cardiovascular events, Haemodialysis, Kidney disease, Mortality rates, Proprotein convertase subtilisin/Kexin type 9

## Abstract

**Background:**

Cardiovascular (CV) disease is the leading cause of mortality in patients with end-stage kidney disease (ESKD). The aim of the present study was to determine whether Proprotein Convertase Subtilisin/Kexin type 9 (PCSK9) could be an independent predictor of CV events and all-cause mortality in black African haemodialysis patients.

**Methods:**

We carried out a prospective cohort study of all consecutive hemodialysis (HD) patients between August 2016 and July 2020, admitted in six hemodialysis centers of Kinshasa, Democratic Republic of Congo. Independent determinants of plasma PCSK-9 measured by ELISA were sought using multiple linear regression analysis. Kaplan-Meier’s method described the incidence of CV events while competitive and proportional risk models looked for independent risk factors for death at the .05 significance level.

**Results:**

Out of 207 HD patients, 91 (43.9%) died; 116 (56.1%) have survived. PCSK9 level was significantly higher in deceased patients compared to survivors: 28.0 (24.0–31.0) ng/l vs 9.6 (8.6–11.6) ng/ml (*p* <  0.001). Patients with plasma PCSK9 levels in tertile 3 had a higher incidence of CV events and mortality compared to patients with plasma PCSK9 levels in tertile 2 or tertile 1 (*p* <  0.001). Tertile 3 negatively influence survival rates (26.6%) compared to tertile 2 (54.7%) and tertile 1 (85.3%). Patients in tertile 3 and tertile 2 had a 4-fold higher risk of death than patients in tertile 1. After adjustment for all parameters, competitive risk analysis showed that mortality was 2 times higher in patients with stroke. Similarly, serum albumin < 3.5 g/dL or PCSK9 in tertile 3 were respectively associated with 2 or 6 times higher rates of deaths.

**Conclusion:**

Elevated plasma PCSK9 level is an independent major predictor of incident CV events and all-cause mortality in black African HD patients.

**Supplementary Information:**

The online version contains supplementary material available at 10.1186/s12882-022-02748-0.

## Background

In chronic kidney disease (CKD) patients not on dialysis, cardiovascular mortality is largely justified by the high level of cholesterol-rich in low-density lipoproteins (LDL-c) [1]. In addition, cardiovascular (CV) events remain the leading cause of death in hemodialysis (HD) patients [2, 3]. However, in those patients, the association between mortality and LDL-c is not yet clearly established. This weakly established relationship is one of the arguments that motivated the international guidelines, the Kidney Disease Improving Global Outcomes (KDIGO), which advise against the initiation of lipid-lowering drugs (statins) in HD patients [4, 5]. These molecules do not effectively reduce the LDL-c level or the resulting mortality [6, 7]. Proprotein Convertase Subtilisin/Kexin type 9 (PCSK9) by inhibiting the recycling of LDL-c receptors and promoting their degradation [8, 9] appears to be the main regulator of plasma levels of LDL-c. PCSK9 is a new marker potentially playing a causative role on CV mortality in maintenance HD patients. However, this association has been rarely evaluated [10]. Therefore, we conducted a cohort study in the HD centers located in Kinshasa, the Democratic Republic of the Congo (DRC) to assess the association between plasma level of PCSK9 and incident CV events as, well all-cause mortality, in Congolese HD patients.

## Methods

### Design and study population

**Fig. 1:**
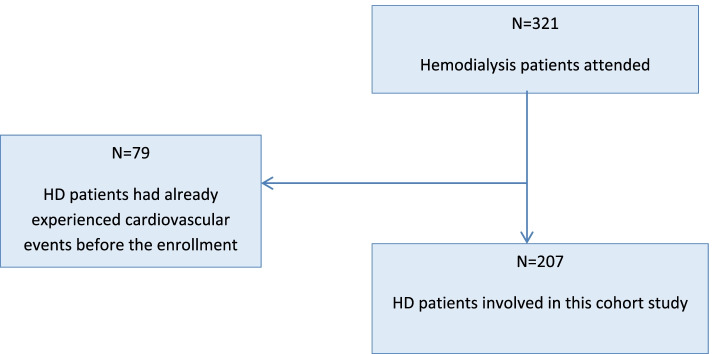
Flowchart describing participants’ sampling process

In this present cohort study, patients receiving hemodialysis (HD) treatment between August 2016 and July 2020 in HD service providers (Kinshasa University Hospital, General Provincial Referral Hospital of Kinshasa; General Referral Hospital of the Congolese National Police; Ngaliema medical Center Clinic; H J Hospitals and AFIA Medical Center of the city of Kinshasa in the Democratic Republic of Congo (DRC) were consecutively enrolled. Inclusion criteria were as follows: being aged at least 16 years with end-stage kidney disease (ESKD) who had been on HD for at least 3 months; receiving three HD sessions a week, and each session lasting 4 h. HD patients having experienced CV events before the enrollment in the present study were excluded (flowchart, Fig. 1). Study subjects were followed up prospectively after baseline assessments. Clinical events were identified, including CV events and death.

### Variables of interest

Variables of interest included: age, sex, cause of death, history of diabetes mellitus, hypertension, smoking, alcoholism, physical activity, hemodialysis vintage, and current medications (in particular statins). Physical examination was carried 15 min before HD and focused on the following parameters: weight (Kg), height (cm), blood pressure (mm Hg), waist circumference (cm), pulse, and heart rate (beat/min). CV events were and recorded on an ad hoc reporting following their manifestations ascertained by the medical team in charge of the patient during the study period. The following biological parameters were recorded: hemoglobin, hematocrit, serum urea, serum creatinine, glycaemia, serum albumin, uric acid, total cholesterol (TC), low-density lipoproteins (LDL-c), high-density lipoproteins (HDL-c), triglycerides (TG), non-HDL-c, calcium, phosphorus, intact parathyroid hormone (PTHi), Vitamin D, PCSK9. Non-biological parameters encompassed: ankle-brachial index (ABI) obtained based on the ratio of the systolic blood pressure (SBP) measured at the ankle to that measured at the arm; the body composition of the study population taken a quarter of an hour before the dialysis session included: sex, age, height, weight, body fat, muscle mass, and BMI. This composition was determined using the scale: OM-BF 214, Brand Omron, Type Body fat monitor, EAN 4015672107045, 2015 with 4 sensor accuracy technology, large LCD panel, 4 user’s memory with guest mode. Overweight and obesity were defined, respectively, by a BMI ≥ 25 kg/m^2^) and ≥ 30 kg/m^2^.

### Laboratory measurements

PCSK9 measurement was performed by the competitive-inhibition enzyme immunoassays using the ELISA MBS920252 kit according to the recommendation from the manufacturer. Detection of the protein ranges from 0.45 ng/ml to 30 ng/ml. For the study, PCSK9 levels were divided into 3 tertiles, respectively: tertile 1, PCSK9 < 9.58 ng/ml (*n* = 69); tertile 2, PCSK9 of 9.58–23.0 ng/ml (*n* = 69) and tertile 3 PCSK9 > 23.0 ng / ml (*n* = 69). The lipid fractions were assayed according to the enzymatic colorimetric method on the Cobas C 311 revised version 2010 automated system. Isolated dyslipidemia was defined as a total cholesterol level ≥ 200 mg/dl; HDL-c < 50 mg/dl in women and < 40 in men; LDL-c ≥ 100 mg/dl or TG ≥ 150 mg/dl [11]. Combined dyslipidemia was defined according to the international Frederickson classification: type I or IV dyslipidemia corresponded to a level of LDL < 100 mg/dl and TG ≥ 150 mg/dl; type IIa: LDL ≥ 100 mg/dl and TG < 150 mg/dl. Type IIb: LDL ≥ 100 mg / dl and TG ≥ 150 mg/dl [12].

### Outcome measures

The primary outcome was incident CV events and death (all-cause of mortality). CV events were defined either as hypertensive heart disease: a constellation of changes in left ventricular hypertrophy caused by high blood pressure; or stroke: refer to symptomatic or silent brain imaging abnormalities, or cardiac failure: ejection fraction > 50%, heart failure with preserved ejection fraction (HFpEF) vs ejection fraction < 50%, heart failure with reduced ejection fraction (HFrEF); or dilated cardiomyopathy: left or biventricular dilatation in the absence of hypertension, valvular disease of coronary artery disease; or myocardial infarction: symptoms of acute myocardial ischemia and ischemia ECG changes or percutaneous angioplasty or coronary bypass surgery, or deep vein thrombosis, or congestive heart failure: functional class III or IV of the New York Heart Association. We counted only the first CV event. All- mortality events from any cause were also considered. The secondary outcome was clinical and laboratory parameters, which were assessed in relation to PCSK9 levels.

### Statistical analysis

Statistical analyses were performed using SPSS version 21 software. Descriptive statistics were presented as the mean plus/minus standard deviation for continuous variables with normal distribution and median (interquartile range or IQR) for continuous data with the non-Gaussian distribution. Absolute and relative frequencies were expressed for categorical variables. Comparisons between groups were made as appropriate, using the Student-t-test (normally distributed variables), Mann-Whitney’s U test (non-normally distributed variables) respectively for continuous variables (quantitative); chi-square, or Fisher’s exact test for categorical (qualitative) variables. In the present study due to the negligible number of missing data, no imputation was performed. Simple linear regression analysis was performed to establish the relationships between lipid profiles and levels of PCSK-9. Independent determinants of PCSK-9 were sought in multiple linear regression. Kaplan-Meier’s method described the incidence of events cardiovascular (time to event CV) from day of dialysis until event CV event and survival (time to death) from the day of dialysis initiation until death or end of the study for patients alive. Patient follow-up was censored at the time of kidney transplantation, loss of follow-up, HD withdrawal, or alive at the end of the study (July 30th, 2020). A Log-rank test was used to compare survival curves (Supplemental Figure S1 A, B, C). Competitive and proportional risk models looked at independent risk factors for death. A variance inflation factor was used to check the presence of multicollinearity between independent variables, and no multicollinearity was detected. Statistical analyses were performed using SPSS version 21 software at the .05 significance level.

The study protocol was approved by the ethics committee of the School of Public Health of the University of Kinshasa (ESP/CE/ 053/2016) and the study was conducted in accordance with the Helsinki principles. All participants signed written informed consent forms before enrollment.

## Results

### Baseline demographic characteristics

A total of 207 HD patients (153 men, mean age 55.4 ± 13.9 years) were included (Table 1). The cumulative incidence of CV events was 47.3%. Comorbid conditions included high blood pressure (87.4%), diabetes (28.0%), hypertensive cardiopathy (16.4%), stroke (15.5%), dilated cardiomyopathy (11.1%), gout (7.7%), myocardial infarction (4.8%), deep vein thrombosis (4.3%) and heart failure (2.4%). The frequency of stroke, hypertensive cardiopathy, and dilated cardiomyopathy was significantly higher in the deceased group compared to surviving group (*p* <  0.001). The causes of death encompassed cardiovascular death (46.1%), infections (21.9%), others [malignancy, malnutrition, electrolyte disorders] (20.9%) and unknown (10.9%). The patients who died at home or between home and hospital were considered to have an unknown cause of death.

**Table 1:** Baseline demographic characteristics and laboratory data

Variables	All *n* = 207	Living *n* = 116	Deceased *n* = 91	*p* value
Age (years)	55.4 ± 13.9	55.9 ± 14.3	54.7 ± 13.4	0.550
Sex				0.403
Male	153 (73.9)	87 (75.0)	66 (72.5)	
Female	54 (26.1)	29 (25.0)	25 (27.5)	
FH-HBP	96 (46.4)	50 (43.1)	46(50.5)	0.177
FH-DM	54(26.1)	29(25.0)	25(27.5)	0.403
FH-KF	10(4.8)	6(5.2)	4(4.4)	0.532
FH-obesity	17(8.2)	11(9.5)	6(6.6)	0.313
FH-gout	4(1.9)	3(2.6)	1(1.1)	0.407
High blood pressure	181(87.4)	100(86.2)	81(89.0)	0.350
Diabetes mellitus	58(28.0)	32(27.6)	26(28.6)	0.499
Gout	16(7.7)	11(9.5)	5(5.5)	0.212
Cardiovascular event	98(47.3)	14(12.1)	84(92.3)	**< 0.001**
Hypertensive cardiopathy	34(16.4)	10(8.6)	24(26.4)	0.001
Stroke	32(15.5)	4(3.4)	28(30.8)	< 0.001
Heart failure	5(2.4)	0	5(5.5)	0.015
Dilated cardiomyopathy	23(11.1)	3(2.6)	20(22.0)	< 0.001
Myocardial infarction	10(4.8)	0	10(11.0)	< 0.001
Deep vein thrombosis	9(4.3)	0	9(9.9)	< 0.001
Physical inactivity	192 (92.8)	104(89.7)	88(96.7)	0.044
Smoking	42(20.3)	25(21.6)	17(18.7)	0.370
Alcohol	100(48.3)	62(53.4)	38(41.8)	0.063
NSAI	40(19.3)	26(22.4)	14(15.4)	0.137
Traditional plants	48(23.2)	22(19.0)	26(28.6)	0.135
Obesity	17(8.2)	10(8.6)	7(7.7)	0.509
Overweight	47(22.7)	32(27.6)	15(16.5)	0.041
Hypercholesterolemia	32(15.5)	21(18.1)	11(12.1)	0.160
Low HDLc	152(73.4)	88(75.9)	64(70.3)	0.231
High LDLc	68(32.9)	38(32.8)	30(33.0)	0.546
Hypertriglyceridemia	61(29.5)	34(29.3)	27(29.7)	0.538
ABI	1.16 ± 0.20	1.16 ± 0.19	1.15 ± 0.20	0.333
< 0.9	8(3.9)	3(2.6)	5(5.5)	
0.9–1.3	144(69.6)	85(73.3)	59(64.8)	
> 1.3	55(26.6)	28(24.1)	27(29.7)	
Radial pulse (bpm)	89.1 ± 13.5	89.1 ± 14.5	89.1 ± 12.2	0.991
BMI (kg/m^2^)	23.4 ± 4.8	23.9 ± 5.2	22.6 ± 4.1	0.042
Fat mass (%)	27.1 ± 9.1	26.6 ± 9.2	27.7 ± 9.1	0.396
Kt/V	1,29 ± 0,17	1,41 ± 0,12	1,14 ± 0,04	< 0,001
Kt/V ≤ 1.26	105 (50.7)	14 (21.1)	91 (100.0)	< 0.001
S. Albumin (g/dL)	3,53 ± 0,70	3,76 ± 0,69	3,22 ± 0,58	< 0,001
S. Albumin < 3.5 (g/dL)	109 (52.7)	42 (36.2)	67 (73.6)	< 0.001

Data are expressed as mean ± standard deviation, absolute (n) proportions or relative frequency (%). *ABI* ankle-brachial index, *BMI* body mass index, *FH-DM* family history of diabetes mellitus, *FH-HBP* family history of high blood pressure, *FH-KF* family history of kidney failure, *NSAIs* non-steroidal anti-inflammatory drugs

### Biological characteristics of the study population

Detailed baseline characteristics are shown in Table 2. In terms of the aforementioned lipid fractions, there was no statistically significant difference between surviving and deceased patients. In contrast, plasma PCSK9 level was significantly higher in deceased patients (mean 28, 95% CI: 24.0–31.0 ng/l) compared to survivors: (mean 9.6 95% CI: 8.6–11.6) ng/ml; *p* <  0.001).

**Table 2:** Baseline biological parameters

Variables	All *n* = 207	Living *n* = 116	Deceased *n* = 91	*p* value
Glycemia fasting (mg/dL)	120.8 ± 49.4	112.3 ± 41,7	129.3 ± 56.1	0.337
Glycemia occasional (mg/dL)	126.4 ± 42.5	151.9 ± 41.7	113.6 ± 40.1	0.223
Hb (mg/dL)	8.6 ± 1.9	8.6 ± 1.8	8.5 ± 1.9	0.673
GB (/mm^3^)	6700.0 (4590.0–9000.0)	6580.0 (4550.0–9000.0)	6925.0 (4567.5–9050.0)	0.444
N (%)	65.0 ± 13.3	63.7 ± 13.7	66.6 ± 12.7	0.192
L (%)	24.9 ± 12.2	25.4 ± 11.6	24.4 ± 12.9	0.611
M (%)	7.0 (4.0–10.0)	7.0 (3.0–10.0)	7.0 (4.0–10.0)	0.448
B (%)	0.0 (0,0–0.05)	0.0 (0.0–0.08)	0.0 (0.0–1.0)	0.302
E (%)	2.0 (1.0–3.0)	1.5 (1.0–3.0)	2.0 (1.0–2.5)	0.585
ESR (mm/1st h)	55.0 (20.0–112.0)	51.0 (20.0–77.0)	42.5 (28.5–85.0)	0.434
CRP (mg/dL)	24.0 (0.0–66.0)	24.0 (1.4–53.8)	36.0 (24.0–48.0)	0.177
S. Creatinin (mg/dL)	9.3 (4.0–16.3)	10.0 (7.5–12.4)	11.3 (6.3–16.3)	0.439
eGFR (ml/min/1.73m^2^)	8.3 (2.9–19.7)	5.9 (5.2–9.4)	7.0 (2.9–11.2)	0.260
Urea (mg/dL)	153.0 (28.2–386.3)	185.2 (112.4–255.3)	235.2 (84.0–386.3)	0.143
Uric Acid (mg/dL)	6.3 (2.7–8.0)	7.2 (5.7–10.2)	6.7 (5.9–8.0)	0.357
TC (mg/dL)	134.5(109.2–271.0)	134.5 (115.5–175.6)	206.3 (141.5–271.0)	0.166
HDLc (mg/dL)	39.1 (19.4–55.0)	39.1 (18.0–48.1)	53.1 (51.2–55.0)	0.854
LDLc (mg/dL)	172.8 (54.6–229.1)	137.0 (75.9–202.9)	127.6 (82.5–172.8)	0.482
TG (mg/dL)	130.4 (53.6–216.0)	103.9 (62.3–131.9)	132.2 (48.4–216.0)	0.897
Total Ca (mmol/L)	2.2 (0.9–2.5)	2.0 (1.7–2.3)	2.3 (5.1–2.5)	0.872
Ionized Ca (mmol/L)	0.87 (0.50–1.29)	0.87 (0.66–1.03)	1.1 (0.98–1.29)	0.557
K (mmol/L)	5.2 (4.2–7.4)	5.1 (4.5–5.8)	6.4 (5.3–7.4)	0.596
Na^+^ (mmol/L)	129.2 ± 9.9	129.7 ± 9.1	128.5 ± 11.1	0.586
Ph (mmol/L)	2.08 (1.83–2.81)	1.97 (1.67–2.55)	2.83 (1.83–2.95)	0.891
Intact PTH (pg/ml)	181.6 (107.0–192.3)	173.6 (86.7–192.3)	190.9 (101.0–192.3)	0.570
Vit D (ng/ml)	52.2 (43.8–63.5)	52.3 (41.1–65.4)	51.8 (36.0–65.7)	0.831
PCSK9 (ng/ml)	17.0 (12.5–20.4)	9.6 (8.6–11.6)	28.0 (24.0–31.0)	< 0.001
Tertile 1 (< 9.58 ng/ml)	68(32.9)	58(50.0)	10(11.0)	
Tertile 2 (9.58–23.0 ng/ml)	75(36.2)	41(35.3)	34(37.4)	
Tertile 3 (> 23.0 ng/ml)	64(30.9)	17(14.7)	47(51.6)	
Anti HVC Antibodies	9(5.2)	3(3.1)	6(8.0)	0.135
Hepatitis B surface antigen	6(3.5)	3(3.1)	3(4.0)	0.526
Anti HIV Antibodies	4(2.3)	1(1.0)	3(4.0)	0.217

Data are expressed as mean ± standard deviation, absolute (n) proportions or relative frequency (%), median and interquartile (range). *ESR* erythrocyte sedimentation rate, *HDL-c* high-density lipoprotein cholesterol, *LDL-c* low-density lipoprotein cholesterol, *MDRD GFR* modification of diet in renal disease-glomerular filtration rate, *PCSK9* Proprotein convertase subtilisin kexin type 9, *TC* Total cholesterol, *TG* triglycerides

### Determinants of PCSK9 in the study population

**Fig. 2:**
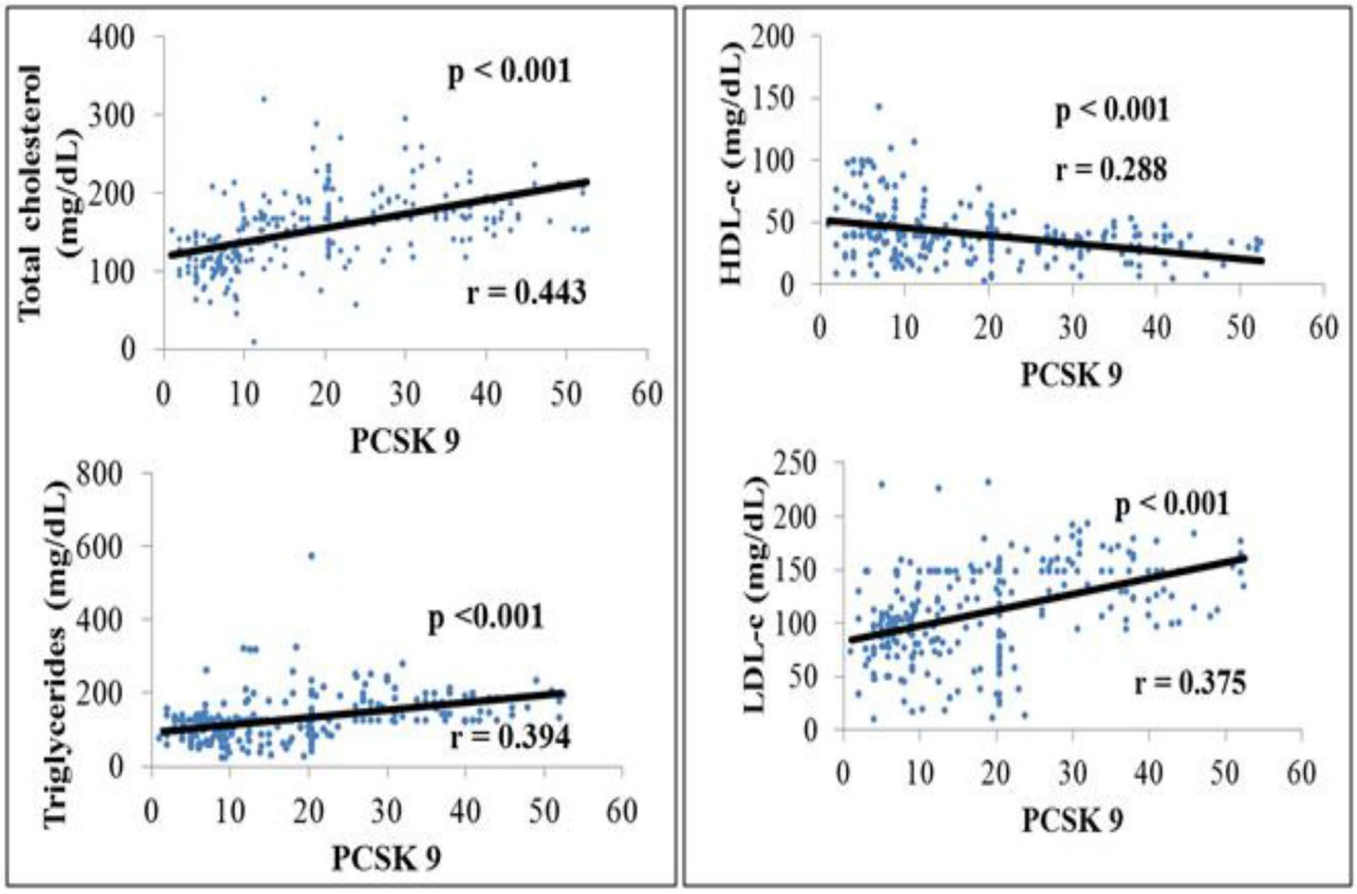
Simple linear correlation between lipid profile and PCSK-9

A positive and significant correlation was found between total cholesterol, triglyceride, LDL-c, and PCSK-9 (Fig. 2). In multivariate analysis, after adjusting for lipid fractions (total cholesterol, triglyceride, HDL-c, and LDL-c), biological (albumin, creatinine, uric acid, and i-PTH) and clinical (ABI) variables, serum albumin, Kt/V, total cholesterol, HDL-c, and triglyceride emerged as independent determinants of PCSK9 level. These parameters explained 52% of the variability of PCSK9 [*R*^2^ = 0.518] (Table 3).

**Table 3:** Determinants factors of PCSK9 in the study population (multiple linear regression)

Variables	Unstandardized β	95% CI	*p*-value
Intercept	93.809	50.85; 136.76	**< 0.0001**
HD duration	0.059	−0.23; 1.35	0.696
Kt/V	−47.316	−70.56; −24.06	**< 0.001**
S. Albumin (g/dL)	−2.360	−7.98; −3.26	**0.009**
S. Creatinine (mg/dL)	−0.079	−0.31; 1.15	0.515
Total Cholesterol (mg/dL)	0.007	−0.08; 0.10	**0.018**
HDLc (mg/dL)	−0.119	−0.30; 0.07	**0.022**
LDLc (mg/dL)	−0.07	−0.10; 1.09	0.885
Triglyceride (mg/dL)	0.010	−0.05; 0.07	**0.015**
i-PTH (pg/mL)	−0.04	−0.02; 1.01	0.608
ABI	2.496	−18.00; 22.99	0.810
	R^2^ = 0.518		

*ABI* ankle-brachial index, *HDL-c* high-density lipoprotein cholesterol, *i-PTH* intact parathyroid hormone, *LDL-c* cholesterol, low-density lipoprotein cholesterol

### Incidence of cardiovascular events based on plasma PCSK9 level

Cardiovascular events increased significantly with PCSK9 tertile (*p* <  0.001). The comparison of the type and incidence of CV events across tertiles shows proportions of dilated cardiomyopathy were significantly higher both in patients at tertile 2 and tertile 3 (*p* = 0.030). By contrast, the incidence of other CV events (e.g. hypertensive heart disease, stroke, heart failure, deep vein thrombosis, myocardial infarction) was not statistically different across tertiles PCSK 9 level (Table 4).

**Table 4:** Cumulative incidence of cardiovascular events according to plasma PCSK9 level

	PCSK9 Tertile 1 *n* = 69	PCSK9 Tertile 2 *n* = 69	PCSK9 Tertile3 *n* = 69	*p* value
All CV Events	13 (18.8)	38 (55.1)	47 (68.1)	**< 0.001**
Hypertensive heart disease	8 (11.6)	10 (14.5)	16 (23.2)	0.194
Stroke	8 (11.6)	11 (15.9)	13 (18.8)	0.532
Heart failure	1 (1.4)	3 (4.3)	1 (1.4)	0.622
Dilated cardiomyopathy	3 (4.3)	7 (10.1)	13 (18.8)	**0.030**
Deep vein thrombosis	1 (1.4)	2 (2.9)	6 (8.7)	0.153
Myocardial infarction	2 (2.9)	3 (4.3)	5 (7.2)	0.612

Data are expressed as absolute (n) proportions or relative frequency (%). *CV* cardiovascular

**Fig. 3:**
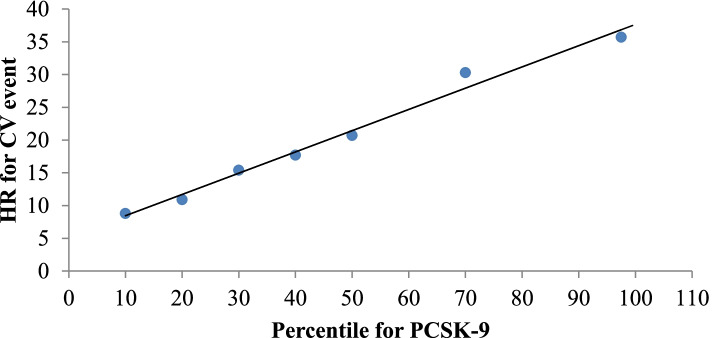
Degree of risk of cardiovascular event by PCSK9 percentile

**Fig. 4:**
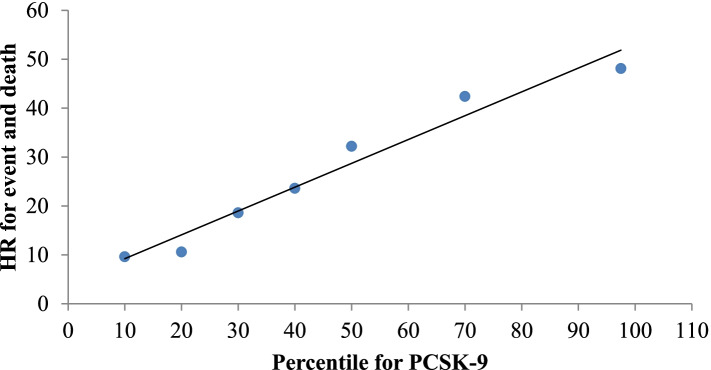
Degree of risk of cardiovascular event and death by PCSK9 percentile

The risk of having a CV event increased linearly with the PCSK9 percentile in the study population (Fig. 3). In contrast, in patients with a CV event, it was observed that as the PCSK9 percentile increased, the risk of death became greater (Fig. 4).

### Survival estimates and predictors of mortality

**Fig. 5:**
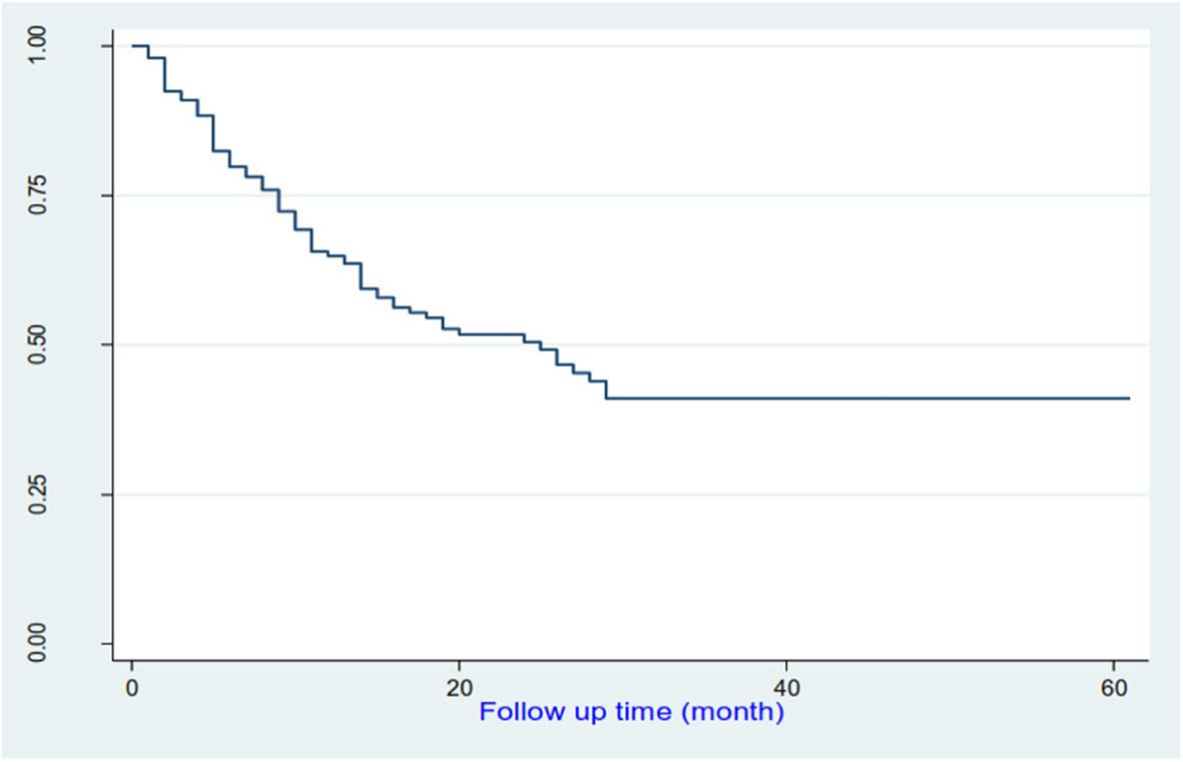
Kaplan-Meier survival estimate of mortality in the study population

During follow-up, 91 deaths (43.9%) and 98 CV events (47.3%) occurred. The overall survival was 80.2% at 6 months; 68.1% at 12 months; 59.4% at 24 months, 56% at 36 months and 56% at 60 months. Median survival was 11.0 (10.0–13.0) months (Fig. 5).

**Fig. 6:**
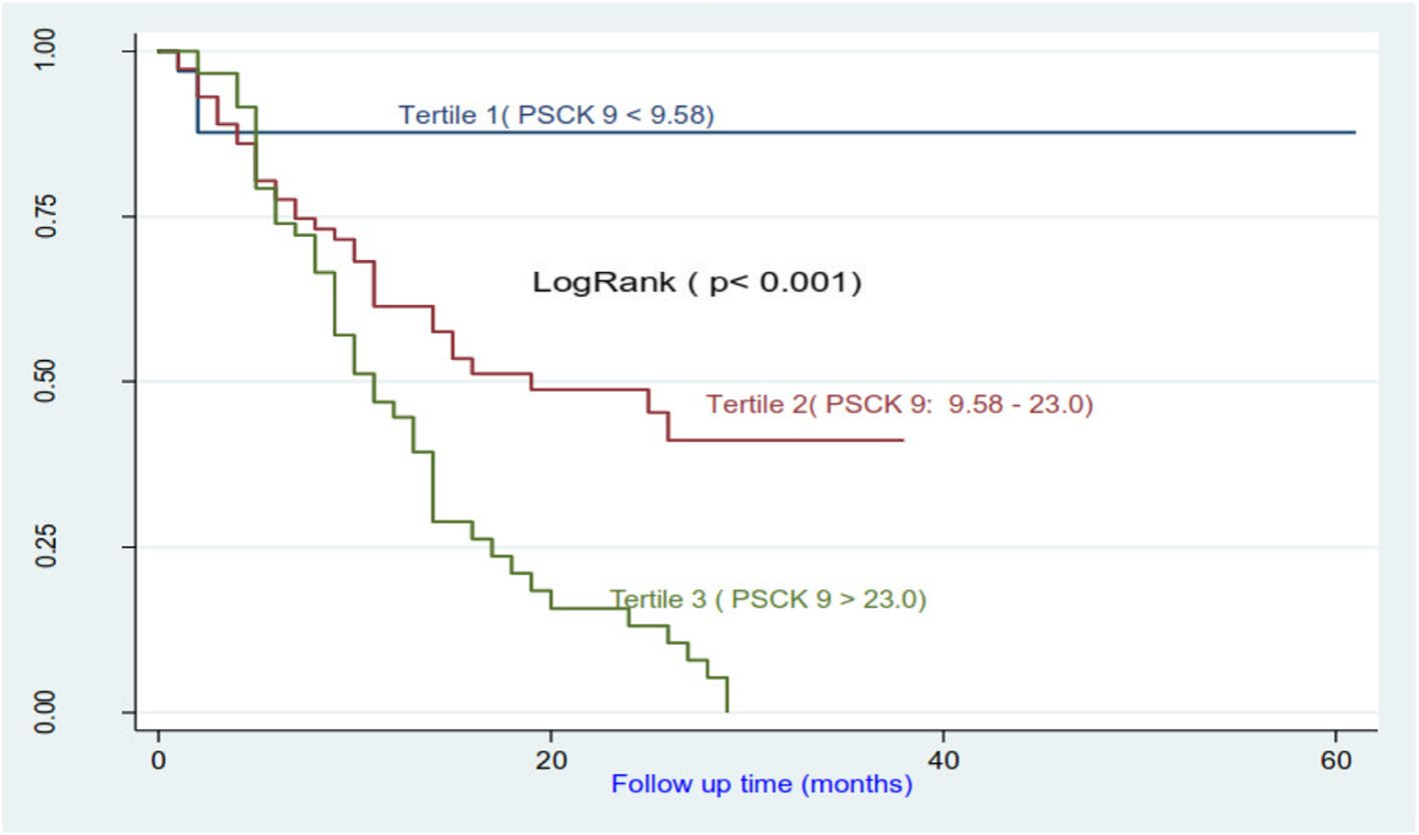
Survival curves in relation to PCSK 9 level

The comparison of survival curves of HD patients according to PCSK9 tertile levels (Fig. 6) showed a highly significant difference (*p* <  0.001). Tertile 3 negatively influence survival (26.6%) compared to tertile 2 (54.7%) and tertile 1 (85.3%).

Table 5 presents the Cox regression and the competitive risk of death analysis. In univariate analysis, HD patients who had an episode of stroke had a 4-fold higher risk of death than those without these conditions. Compared to patients with serum albumin level ≥ 3.5 g/dl, patients with serum albumin level < 3.5 g/dl had 3 times the probability of death. According to PCSK9 tertiles, patients in tertile 3 and tertile 2 had a 4-fold higher risk of death than patients in tertile 1. After adjustment for all parameters and using competitive risk analysis, mortality was 2 times higher in patients with stroke. Similarly, serum albumin < 3.5 g/dl or PCSK9 in tertile 3 was associated with 2 or 6 times higher rates of death (Table 5).

**Table 5:** Risk factors for death in the study population (Cox Regression Model and competitive risk)

		Univariate analysis		Cox Regression		Competitive Risk	
Risk factors	cumulative incidence of death % (95% CI)	HR (95%CI)	*P* value	Adjusted HR (95% CI)	*P* value	Adjusted HR (95% CI)	*p* value
Stroke							
-No	0.53 (0.44–0.65)	1		1		1	
-Yes	0.88 (0.77–0.99)	3.59 (2,29-5,63)	< 0.001	2.50 (1.56–4.00)	< 0.001	1.67 (1.06–2.67)	0.001
S. Albumin (g/dL)							
≥ 3.5	0.35 (0.30–0.41)	1		1		1	
< 3.5	0.65 (0.61–0.76)	2.96 (1.86–4.73)	< 0.001	2.07 (1.28–3.35)	0.030	1.76 (1.06–2.91)	0.027
PCSK 9							
-Tertile 1	0.15 (0.08–0.26)	1		1		1	
-Tertile 2	0.59 (0.46–0.76)	4.06 (2.00–8.26)	< 0.001	3.20 (1.58–6.50)	0.001	3.93 (2.63–5.23)	< 0.001
-Tertile 3	0.97 (0.94–1.00)	8.33 (4.16–16.72)	< 0.001	5.72 (2.88–11.35)	< 0.001	6.47 (5.19–7.75)	< 0.001

*CI* Confidence Intervalle, *PCSK9* Proprotein convertase subtilisin kexin type 9, *HR* Hazard ratios

## Discussion

This prospective observational study showed for the first time that the incidence of CV events increases with plasma PCSK9 level in black African HD patients. We found also that Kt/V, serum albumin, total cholesterol, triglyceride, and HDL-C, were independent determinants of PCSK9 in HD patients without statin treatment. These parameters alone accounted for more than half of the fluctuations in PCSK9 levels. Patients with plasma PCSK9 levels at tertile 3 had a high incidence of CV events and mortality. This risk of CV event and subsequent mortality increased linearly with the PCSK9 level. In addition, high PCSK9 levels negatively influenced the survival of the patients in the study. These observations suggest that PCSK9 may be a predicting biomarker for CV event occurrence in the hemodialysis patient population. While total cholesterol, triglyceride, HDLc were independent determinants of PCSK9 level in this study, Hwang HS et al. [10] studying HD patients on statin treatment identified no association between PCSK9 and lipid fractions (total cholesterol, LDL, HDL-c, triglyceride). The discrepancy in results between the two studies may be due in part to the fact that the HD patients in the present study were not on statin therapy and the hemodialysis treatment was not adequate given the fact that Kt/V is an independent determinant of PCSK9 (giving the preponderance of HD patients having Kt/V < 1.21, 51%). Indeed, both statin treatment and adequate HD-induced dysregulation in lipid parameters and the effect of HD on circulating PCSK9 should normally decrease the strength of this relationship [6, 10, 13, 14]. Hypoalbuminemia is associated both with elevated PCSK9 levels and all-cause mortality in the present study. The exact mechanism of hypoalbuminemia–associated with elevated PCSK9 levels remains unknown but might be due to increased secretion of PCSK9 from hepatocytes coupled with decreased clearance encountered in nephrotic syndrome [15]. It is likely that most HD patients in this study exhibit proteinuria given the preponderance of primitive or secondary glomerulonephritis (diabetes, HVC, HIV, and HVB infection). Yet, this study did not address the specific causes of albuminuria in individual subjects.

HD patients at tertiles 2 and 3 of PCSK9 had a higher risk of death than those in tertile 1. This association remained significant after adjusting for all risk factors for death in the patients included in this cohort. A similar association was observed at the beginning of HD in Korean patients with a history of cardiovascular disease and under lipid-lowering treatment [16]. Yet, our patients were not on lipid-lowering therapy; both family and personal history of cardiovascular risk did not show any significant difference between surviving and deceased patients. The association of PCSK9 and mortality in HD patients appeared to be explained by the fact that the gene encoding PCSK9 in CKD patients can undergo mutations [17]. Indeed, in animal models, a gain in the function of PCSK9 is associated with an increase in levels of fasting plasma triglycerides in mice [18]. Conversely, a loss of PCSK9 function corresponded to a marked reduction in postprandial triglyceride levels in mice [19]. Furthermore, PCSK9 loss-of-function mutations have been associated with lower LDL-c levels in patients with ESKD and reduced cardiovascular risk [20, 21]. Therefore, it is likely that our patients may have a gain in the function of PCSK9. As the gain of function is crucial for the catabolism of LDL-c receptors, LDL-c levels increase. LDL-c contains ApoB particles rich in VLDL-c and triglycerides, are very sensitive to oxidation, and are very atherogenic [22]. Indeed, an interaction between lipoproteins and the immune system creates atheromatous plaques in the vascular wall [23, 24]. A positive correlation between plasma PCSK9 levels and carotid calcifications has also been demonstrated by CT scans in patients with familial hypercholesterolemia [25]. Thus, the high plasma level of PCSK9 and the subsequent atherosclerosis process may explain, at least partly, the high cardiovascular mortality rate observed in HD patients studied [26, 27].

Patients in the present study were not on statin treatment. Indeed, statins often prescribed in CKD increase the circulating level of PCSK9 by activating a binding protein Sterol Regulatory element-binding Protein-2 [SREBP-2] [14]. The high level of PCSK9 observed in our study implies a higher CV event [28] which in turn may justify the higher mortality in these patients.

In addition, PCSK9 is synthesized as a precursor of about 72 kDa which must undergo autocatalytic cleavage between the catalytic pro-domain and domain within the endoplasmic reticulum of the hepatocyte before being secreted into the bloodstream [29]. This molecular weight makes PCSK9 a large molecule that theoretically is not filtered by conventional HD high flux. Conventional filters are effective for molecules with a molecular weight less than 65 kDa. Therefore, high levels of circulating PCSK9 might be due to a lack of dialyzability with high-flux, conventional HD. This finding suggests that hemofiltration or the use of High Flux filters could be beneficial in this setting in order to reduce C-V deaths in dialysis patients. Alternatively, these high PCSK9 levels may also be due to the inflammatory nature of kidney failure often observed in maintenance HD patients [15, 30].

The present study showed a high incidence of CV events and mortality in haemodialysis patients with PCSK9 in tertile 3. The risk of CV events and mortality increased linearly with PCSK9 level while the levels of plasma lipid fractions remained without significant differences between the survivors and the deceased patients. The current finding is consistent with previous studies showing that PCSK9 contributes to the development of CV events independently of traditional CV risk factors [10]. The level of PCSK9 could therefore be a new biomarker of CV risk in HD patients.

Strengths of this study include the concomitant measurement of PCSK9 and lipid fractions in HD patients attending multicenter of HD in Kinshasa and the investigation of the implications of their association on CV events as well as the survival of these patients. Finally, several variables were included in the analysis including laboratory parameters and HD session associated variables.

Limitations are related to the methodological approach. Firstly, measurement of PCSK9 and lipids was only performed once at the beginning of the study. Secondly, PCSK9 activity would be more accurate if PCSK9 has been measured bound to LDL-c receptors. Thirdly, the type of vascular access was not considered in the analysis, the variable which could have some impact on survival. Finally, our study is also based on relatively small sample size.

## Conclusion

In this study, elevated plasma PCSK9 levels independently predicts incident CV events and all-cause mortality in HD black African patients. Future studies are needed to determine the genetic mutations of PCSK9 in the black African hemodialysis patient population.

## Supplementary Information

**Additional file 1.**

## Data Availability

The data and analyses carried out for this study are available from the corresponding author: francoiskajingulu@gmail.com
