# Supplementary figures and images for: Circulating Proprotein Convertase Subtilisin/Kexin type 9 level independently predicts incident cardiovascular events and all-cause mortality in hemodialysis black Africans patients
Source: BMC Nephrol. 2022 Mar 30;23:123. doi: 10.1186/s12882-022-02748-0 (PMC8969257; doi:10.1186/s12882-022-02748-0)

Fig S1. Cumulative survival using Kaplan Meier curve


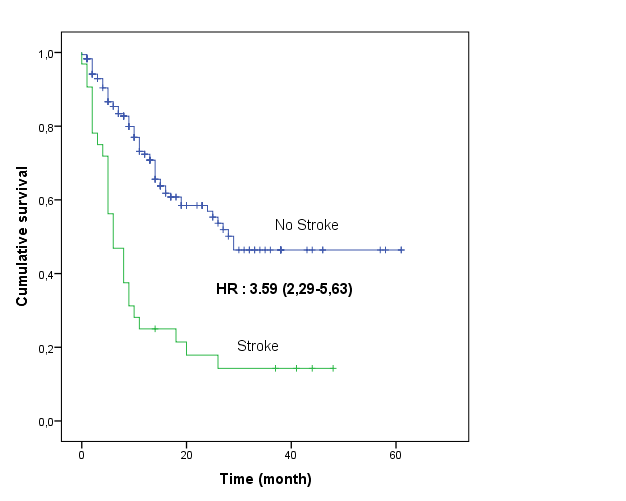

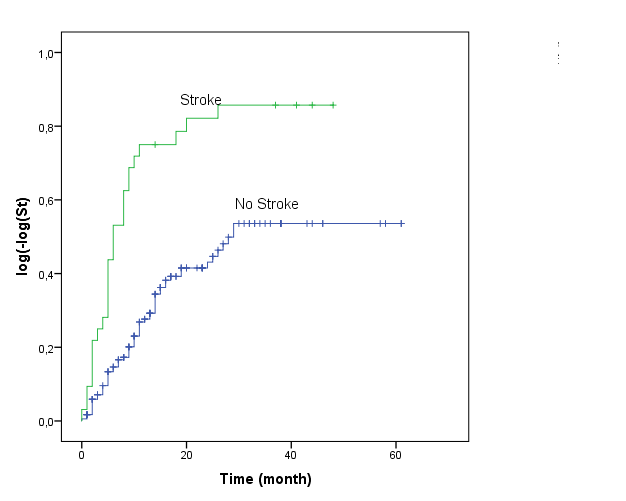


Fig S 1A


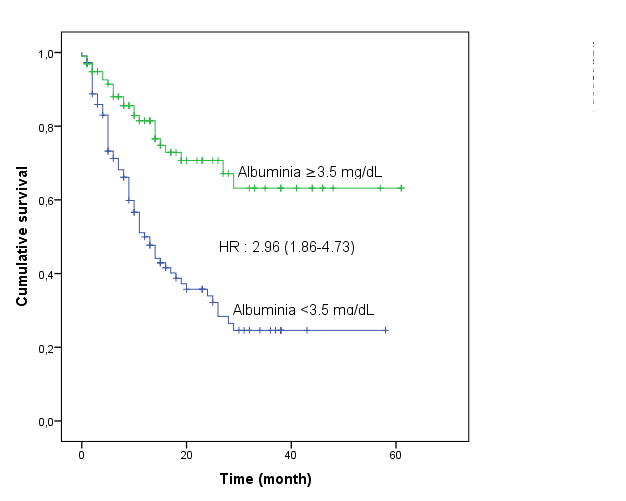

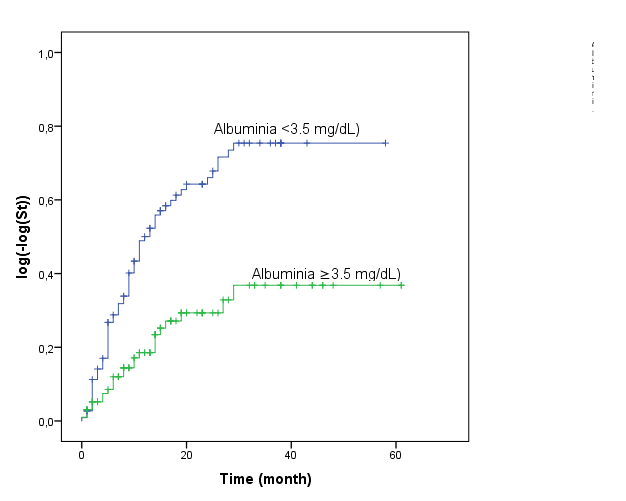


Fig S 1B


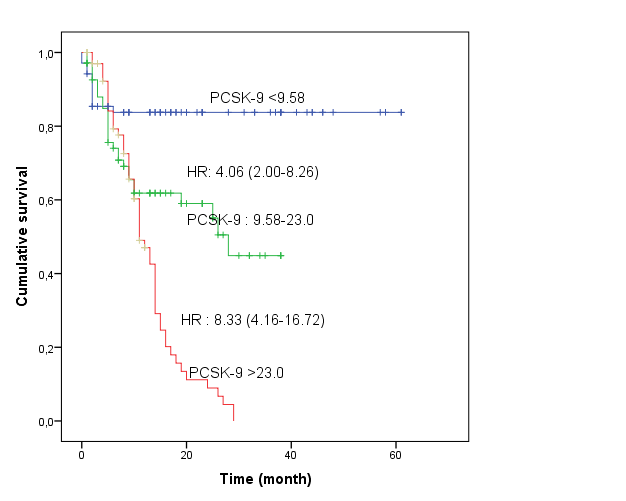

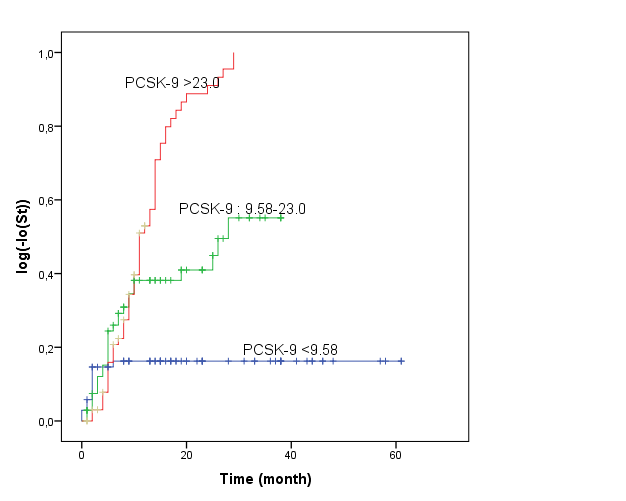


Fig S1C.

Supplement: Supplementary file 1 — Additional file 1. [file 12882_2022_2748_MOESM1_ESM.docx]
